# Taking control: reorganization of the host cytoskeleton by *Chlamydia*

**DOI:** 10.12688/f1000research.12316.1

**Published:** 2017-11-29

**Authors:** Jordan Wesolowski, Fabienne Paumet

**Affiliations:** 1 Department of Microbiology and Immunology, Thomas Jefferson University, Philadelphia, PA, 19107, USA

**Keywords:** Chlamydia, cytoskeleton, actin, microtubules, GTPase, ARF, post-translational modification, pathogenicity

## Abstract

Both actin and microtubules are major cytoskeletal elements in eukaryotic cells that participate in many cellular processes, including cell division and motility, vesicle and organelle movement, and the maintenance of cell shape. Inside its host cell, the human pathogen *Chlamydia trachomatis *manipulates the cytoskeleton to promote its survival and enhance its pathogenicity. In particular, *Chlamydia* induces the drastic rearrangement of both actin and microtubules, which is vital for its entry, inclusion structure and development, and host cell exit. As significant progress in *Chlamydia* genetics has greatly enhanced our understanding of how this pathogen co-opts the host cytoskeleton, we will discuss the machinery used by *Chlamydia* to coordinate the reorganization of actin and microtubules.

## Introduction

The *Chlamydiaceae* constitute a family of obligate intracellular bacteria that encompasses numerous species. With ~92 million new cases/year worldwide, *Chlamydia trachomatis* is the most frequent cause of bacterial sexually transmitted infections and is the leading cause of preventable infectious blindness called trachoma ^1– 3^. Trachoma is a significant problem in the developing world, where access to healthcare is limited and antibiotics are scarce. *Chlamydia* infections are also associated with chronic diseases and increased risk for cervical cancer ^4, 5^, making this infection a significant socioeconomic and medical burden in both developed and developing countries.

**Figure 1.**
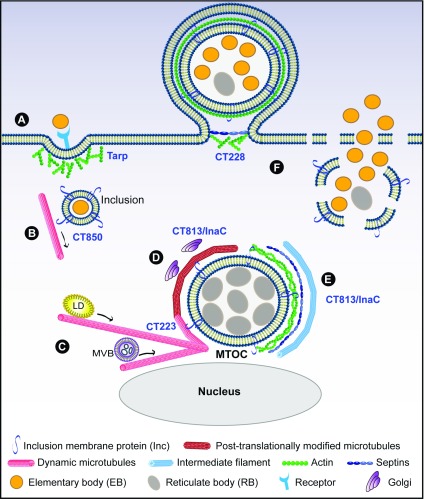
Reorganization of the host cytoskeleton during *Chlamydia trachomatis* infection. ( **A**) Entry during which a translocated actin-recruiting phosphoprotein (Tarp) induces actin polymerization; ( **B**) transport of the nascent inclusion to the microtubule-organizing center (MTOC) using CT850; ( **C**) formation of microtubule cages around the inclusion, in which CT223 is likely involved, and microtubule-dependent movement of lipid droplets (LDs) and multi-vesicular bodies (MVBs) towards the inclusion; ( **D**) post-translational modifications of microtubule cages and positioning of Golgi mini-stacks around the inclusion controlled by CT813/InaC; ( **E**) structural scaffolds of actin, septins, and intermediate filaments reinforce the growing inclusion membrane in a CT813-dependent manner; ( **F**) *Chlamydia* exits the host cell using CT228-dependent extrusion (left) or through cell lysis (right).

Chlamydiae exhibit a unique biphasic life cycle, cycling between a metabolically inactive but infectious small elementary body (EB ~200 nm) and a noninfectious metabolically active and dividing large reticulate body (RB ~800 nm) ^6^. *Chlamydia* spends the majority of its life as an intracellular RB. Soon after invasion, EBs differentiate into RBs and replicate within a membrane-bound compartment called an “inclusion”. This obligate intracellular lifestyle required *Chlamydia* to develop an effective strategy to manipulate host cell pathways in order to ensure its survival and replication. Among the many pathways that *Chlamydia* co-opts, a common and intriguing target for all *Chlamydia* species has emerged: the host cytoskeleton ^7^. We will discuss the recent advances concerning the role played by the host cytoskeleton in the growth and structural maintenance of the chlamydial inclusion (see Figure 1).

## *Chlamydia trachomatis* recruits actin to enter its host cell and uses microtubules to travel to the microtubule-organizing center

Because of their obligate intracellular nature, Chlamydiae have evolved very efficient ways to enter eukaryotic cells ( Figure 1A). During infection, EBs attach to the host cell surface via a relatively weak electrostatic interaction with heparan sulfate moieties ^8^. Then a stronger and more specific binding to a cellular receptor takes place, during which the majority of the EBs are internalized via an actin-dependent event ^9^. While a number of receptors have been identified, including PDGFRβ, β1-integrin, and Ephrin A2, depletion of a single receptor is not sufficient to block entry, suggesting that *Chlamydia* utilizes more than one receptor to invade its host cell ^10, 11^.

To promote entry into non-phagocytic epithelial cells, *C. trachomatis* delivers a translocated actin-recruiting phosphoprotein (Tarp, also known as CT456) ^12– 14^ into the host cytoplasm ( Figure 1A). In a phosphorylation-dependent manner, Tarp recruits guanine nucleotide exchange factors that activate Rac1, a member of the Rho family of GTPases ^9, 10, 13, 15^. Whereas *Chlamydia caviae* uses both Rac1 and Cdc42 to promote its entry, *C. trachomatis* recruits only Rac1, and not Cdc42 or RhoA ^16, 17^. Hijacking only one Rho GTPase family member is unique to *C. trachomatis*, as other intracellular bacteria including *Salmonella* and *Shigella* usually use multiple isoforms such as RhoA and Cdc42 ^18– 20^. In addition to its Rac1 recruitment function, Tarp also directly binds to actin monomers and nucleates new unbranched actin filaments ^21, 22^. These linear filaments are then branched via the host Arp2/3 complex, which is activated by the Rac1 signaling pathway ^9, 13^. Thus, Tarp functions as both an actin nucleator and a signaling platform to locally remodel the actin cytoskeleton and promote *Chlamydia* invasion.

Nascent *C. trachomatis*-containing inclusions then use the minus-end-directed microtubule motor dynein to move from the cell periphery to the microtubule-organizing center (MTOC), where the inclusion resides for the duration of the life cycle ^23^ ( Figure 1B). This is a pathogen-driven event in which the inclusion protein CT850 is involved through its interaction with the dynein light chain DYNLT1 ^24^. At the MTOC, Src family kinases control the tight association between inclusions and centrosomes ^25^. Additional inclusion proteins including IncB, CT101, and CT222 are concentrated at these contact points between inclusions and centrosomes, suggesting their potential contribution to the transport of the inclusion ^24^. In fact, during *Chlamydia psittaci* infection, IncB has been shown to interact with Snapin, which also binds dynein, thus connecting the inclusion to the microtubule network ^26^. The association of inclusions with the MTOC is a common characteristic for a number of *Chlamydia* species, suggesting that this event is essential for *Chlamydia*’s life cycle. One possibility is that the MTOC brings host organelles and chlamydial inclusions in close proximity, thus facilitating the transfer of nutrients and lipids from the host to the inclusion. Additionally, the clustering of the inclusions at the MTOC is necessary for the homotypic fusion of inclusions to take place during *C. trachomatis* infection, as the dissociation of the inclusions from the MTOC inhibits this fusion event ^27^. Homotypic fusion is critical for *C. trachomatis* pathogenicity, as non-fusing mutants grow significantly slower than their wild-type counterparts ^28, 29^. In particular, *C. trachomatis* strains that do not undergo homotypic fusion are also replication-defective and cause significantly milder disease in humans ^28– 30^. Given the importance of microtubule-based transport of the inclusion in *Chlamydia* development, additional unidentified *Chlamydia* effectors are likely involved in this process.

## *Chlamydia* creates microtubule cages to support the development of its inclusion

Around 12 hours post-infection (PI), microtubules likely assemble around the inclusion under the control of the chlamydial effector CT223/IPAM, which has been shown to alter microtubule organization through the host centrosomal protein of 170 kDa (CEP170) in transfected cells ^31^ ( Figure 1C). There, microtubules encasing the inclusion are stabilized, which allows them to undergo post-translational modifications (PTMs), including detyrosination and acetylation ^32, 33^ ( Figure 1D). These PTMs influence microtubule structure and depolymerization rates ^34^ and have been implicated in the relocation of the Golgi apparatus around the chlamydial inclusion ^32, 33^. In most cells, dynamic microtubules have a half-life of about 5–10 minutes, while modified microtubules can persist for hours ^35^, suggesting that *Chlamydia* uses these PTM microtubules to establish a stable long-term relationship with its host. Detyrosination is the best-characterized modification and involves the removal of the carboxy-terminal tyrosine from α-tubulin by tubulin carboxypeptidase, thus exposing a glutamic acid as the new C-terminus ^36^.

Interestingly, stable microtubules are involved in the repositioning of organelles. During infection, the Golgi is a major source of host lipids, including sphingomyelin and cholesterol ^37– 40^. To enhance access to these lipids, *Chlamydia* induces the fragmentation of the Golgi into mini-stacks, which are then recruited around the inclusion in a microtubule-dependent manner ^32, 41^ ( Figure 1D). In addition to controlling Golgi positioning, detyrosinated microtubules are involved in other trafficking events, including the recycling of endocytosed transferrin ^42^ and the dispersal of lipid droplets ^43^, which are also co-opted by *Chlamydia* ^39, 44^. Lipid droplets and multi-vesicular bodies are redirected towards the chlamydial inclusion along microtubules to provide fatty acids, which are important for *Chlamydia* replication ^39, 40, 44, 45^ ( Figure 1C). The endoplasmic reticulum (ER) has also been associated with stable microtubules, in particular acetylated microtubules, along which ER tubules slide ^46^. *Chlamydia* hijacks the ER and promotes the formation of ER-inclusion contact sites, which are important for the transfer of lipids to the inclusion ^47– 49^. During *Chlamydia* infection, the acetylated microtubules that surround the inclusion could enable the ER to slide towards the inclusion, thus allowing IncD/CERT to interact with the ER-resident proteins VAPA/VAPB and form ER-inclusion contact sites ^47, 48^. Although treatment with nocodazole, which disrupts microtubules, failed to prevent ER accumulation around the inclusion ^49^, acetylated microtubules are notoriously resistant to nocodazole treatment and may still be present following treatment.

Recently, we have shown that the chlamydial protein CT813 (also called InaC ^50^) is critical for promoting microtubule modifications during infection through its interaction with and activation of the small host GTPases ARF1 and ARF4 ^33^, supporting prior data showing interactions between CT813 and ARF proteins ^50^ ( Figure 1D). It is unclear how the CT813:ARF complex is able to influence microtubule PTMs. However, inhibitors of RhoA and ROCK (Rho-associated protein kinase) decrease the number of inclusions associated with stable microtubules, suggesting that both of these proteins are involved in this process ^32^. There is evidence demonstrating that RhoA triggers microtubule stabilization via its interaction with the mammalian homolog of Diaphanous (mDia). The activation of mDia generates capped microtubules, thus preventing catastrophic microtubule disassembly ^51, 52^. Interestingly, DIAPH2 was identified in an RNAi screen in Drosophila S1 cells as important for chlamydial inclusion development ^10^. It would be interesting to determine whether *C. trachomatis* co-opts mDia to stabilize microtubules and generate the post-translationally modified microtubule cages around the inclusion, particularly since mDia is also involved in actin polymerization ^53^.

## *Chlamydia trachomatis* builds actin scaffolds around its inclusion to promote inclusion stability

As the inclusion continues to grow, actin and intermediate filaments associate with the inclusion ^54^ ( Figure 1E). This association increases progressively from ~20 hours PI until the end of *Chlamydia*’s intracellular life cycle. Disruption of the actin cytoskeleton results in the rupture of the inclusion membrane and the leakage of *C. trachomatis* into the host cytoplasm, demonstrating that the maintenance of the inclusion’s integrity requires intact actin cages ^54^. Interestingly, RhoA—but not ROCK—also plays a major role in this event, as it is recruited to the inclusion and its depletion results in a substantial loss of actin scaffolds around the inclusion ^54^.

The chlamydial protein CT813 is the only effector identified to date to regulate actin recruitment around the inclusion ^33, 50^ ( Figure 1E). Interestingly, this function of CT813 appears to be independent of its role in regulating post-translationally modified microtubule cages, as the overexpression of CT813 in wild-type *Chlamydia* results in the loss of post-translationally modified microtubules but not actin cages ^33^. Together, these data suggest that both CT813 and RhoA participate in actin cage formation and microtubule stabilization. While microtubule stabilization also depends on ARF1/ARF4 and ROCK, actin polymerization does not require ROCK. The role of ARF in the formation of actin cages remains unclear, as depletion of ARF does not affect actin polymerization ^33^.

Recently, the actin cytoskeleton has been implicated in Golgi reorganization during infection. Using chemical mutagenesis, it has been suggested that CT813 organizes Golgi mini-stacks around the inclusion through the formation of actin cages ^50^. However, a CT813-overexpressing *Chlamydia* strain that has actin but no post-translationally modified microtubule cages displays a defect in Golgi organization around the inclusion. This suggests that it is the CT813-dependent induction of post-translationally modified microtubule cages that controls Golgi organization ^33^. Therefore, the exact role of the actin cytoskeleton in organelle repositioning during *Chlamydia* infection is still unclear.

## *Chlamydia* uses the actin cytoskeleton to exit host cells by extrusion

*Chlamydia* exits the host cell through two mutually exclusive mechanisms: extrusion and cell lysis ( Figure 1F, left and right, respectively). Note that to exit their host cells using the lytic process, *Chlamydia* must extricate themselves from the cytoskeleton structures that encase the inclusion, in particular the actin scaffold ^55^. Pgp4, a transcription factor encoded by the chlamydial plasmid, is essential for actin depolymerization prior to cell exit, as the deletion of this regulatory gene prevents actin disassembly and completely blocks *Chlamydia* exit ^55^. The chlamydial protease activity factor, which cleaves intermediate filaments, is also involved in cell exit, but it does not play a role in actin disassembly ^54– 56^.

While the lytic pathway requires actin depolymerization to proceed, extrusion is regulated by an acto-myosin-mediated mechanism ^57, 58^. Extrusion involves the protrusion of the intact membrane-bound inclusion out of the cell and the pinching off of the inclusion into a separate compartment. The resulting extruded inclusion is surrounded by the actin cytoskeleton ^59^, the host plasma membrane, and a thin layer of cytoplasm between plasma and inclusion membranes ^57^. Extrusion requires N-WASP-mediated actin polymerization and myosin II-dependent contraction of stable actin filaments ^57, 58^. The septin family of cytoskeletal proteins regulates actin fiber formation on the inclusion membrane ^60^ through an unknown mechanism. Interestingly, RhoA is also involved in this process, where it specifically regulates the final stage of extrusion—pinching off and separation of the extrusion from the host cell ^57^.

The signals that dictate whether *Chlamydia* exits the host cell by lysis or extrusion are not well understood. However, the Chlamydial inclusion protein CT228 has been shown to play a central role in this process ^58^. CT228 recruits the MYPTI subunit of myosin phosphatase to microdomains on the inclusion membrane early during infection. MYPTI-mediated phosphorylation of myosin light chain II (MLC2) favors extrusion-mediated exit, while the depletion or dephosphorylation of MLC2 shifts the balance towards the lytic pathway. Thus, *Chlamydia* establishes local cytoskeletal signaling networks on the inclusion membrane to direct its escape from the host cell.

## Concluding remarks

*Chlamydia* has evolved efficient mechanisms to hijack essential components of the host cytoskeleton. The successful establishment of *Chlamydia*’s intracellular niche and dissemination of infectious progeny relies on the proper spatial and temporal control of both actin and microtubules. To orchestrate this balance, *Chlamydia* employs effector proteins to recruit host proteins to the inclusion membrane and modulate the activity of host cytoskeletal signaling networks. Recent work has only begun to shed light on the identity of these chlamydial effector proteins.

Almost a decade ago, an RNAi screen in *C. trachomatis*-infected Drosophila cells revealed the importance of numerous host cytoskeleton-associated proteins in inclusion development, supporting the critical role of the cytoskeleton during infection ^10^. However, limited mechanistic information regarding the role of chlamydial effector proteins in this process was available owing to the intractability of *Chlamydia* to genetic manipulation. Recent advances in *Chlamydia* genetics and the expansion of the *Chlamydia* genetic toolbox now provide the tools necessary to dissect the molecular pathways and the chlamydial effectors that control the interactions between the chlamydial inclusion and the host cytoskeleton. Identifying these mechanisms is important not only for understanding *Chlamydia* pathogenesis and developing novel therapeutics but also because it has the potential to identify new host cellular pathways that regulate the cytoskeleton.
